# Supplementary material for: KRas-transformed epithelia cells invade and partially dedifferentiate by basal cell extrusion
Source: Nat Commun. 2021 Dec 10;12:7180. doi: 10.1038/s41467-021-27513-z (PMC8664939; doi:10.1038/s41467-021-27513-z)
Supplement: Supplementary file 1 — Supplementary Information [file 41467_2021_27513_MOESM1_ESM.pdf]

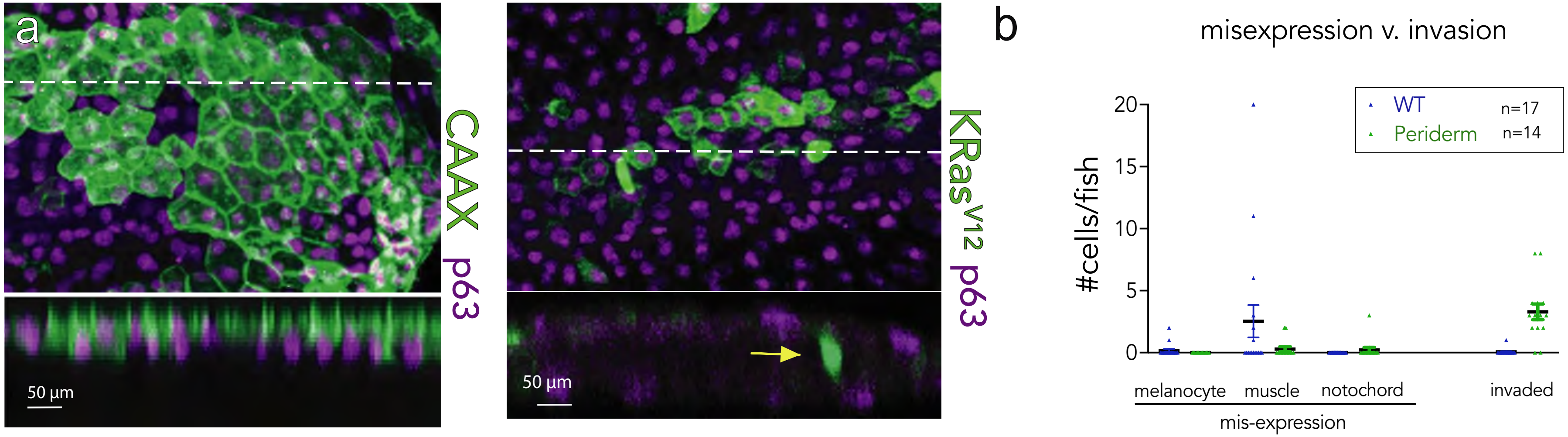

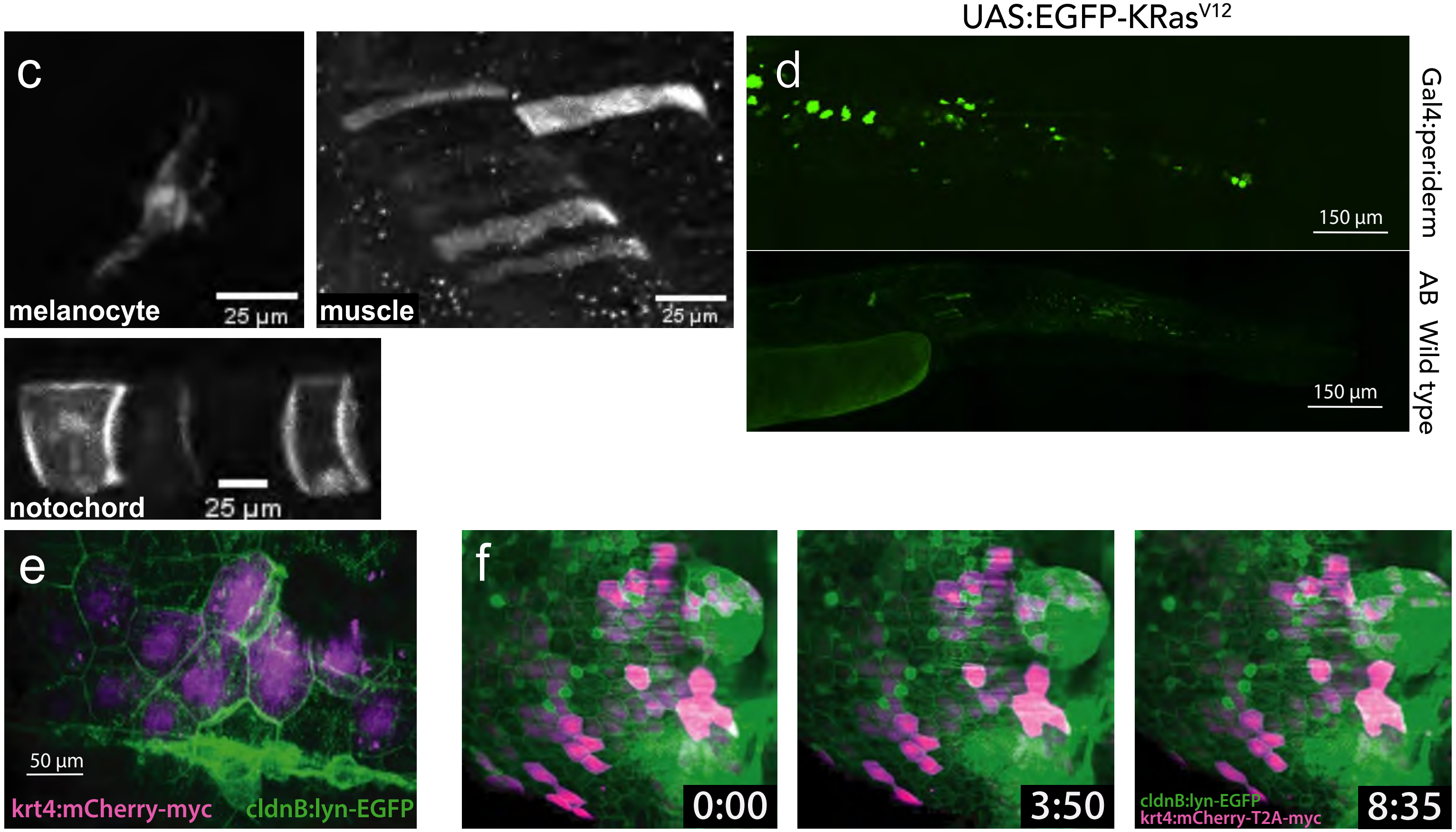

Supplementary Fig.1. Only EGFP-KRas^{V12} cells internalize in wild-type embryos. (a) Maximum intensity and XZ projections (below) of EGFP-CAAX- or dt-KRas^{V12}-injected wild-type 48 hpf embryos, with arrow indicating a KRas^{V12} cell internalized underneath p63+ basal keratinocytes. (b) Number of cells per fish tail misexpressing GFP in notochord, muscle, and melanocytes ± SEM, compared to number of invaded cells (scored as internal cells minus mis-expressing cells) following injection of UAS:GFP-KRas^{V12} into WT or Gal4:periderm lines. (c) Examples of typical mis-expressing cell types in notochord, muscle, or melanocyte, scored in (B). (d) Example of tails from UAS:GFP-KRas^{V12} expressed in WT or Gal4:periderm lines, scored in (B). (e) Maximum intensity projection of krt4:mCherry-T2A-cMyc cells in a cldnB:lyn-EGFP reporter line, representative of 3 embryos. (F) Stills from Movie S6 (hh:mm) showing that cMyc cells do not extrude or invade, where n=14 embryos.Source data are provided as a Source Data file

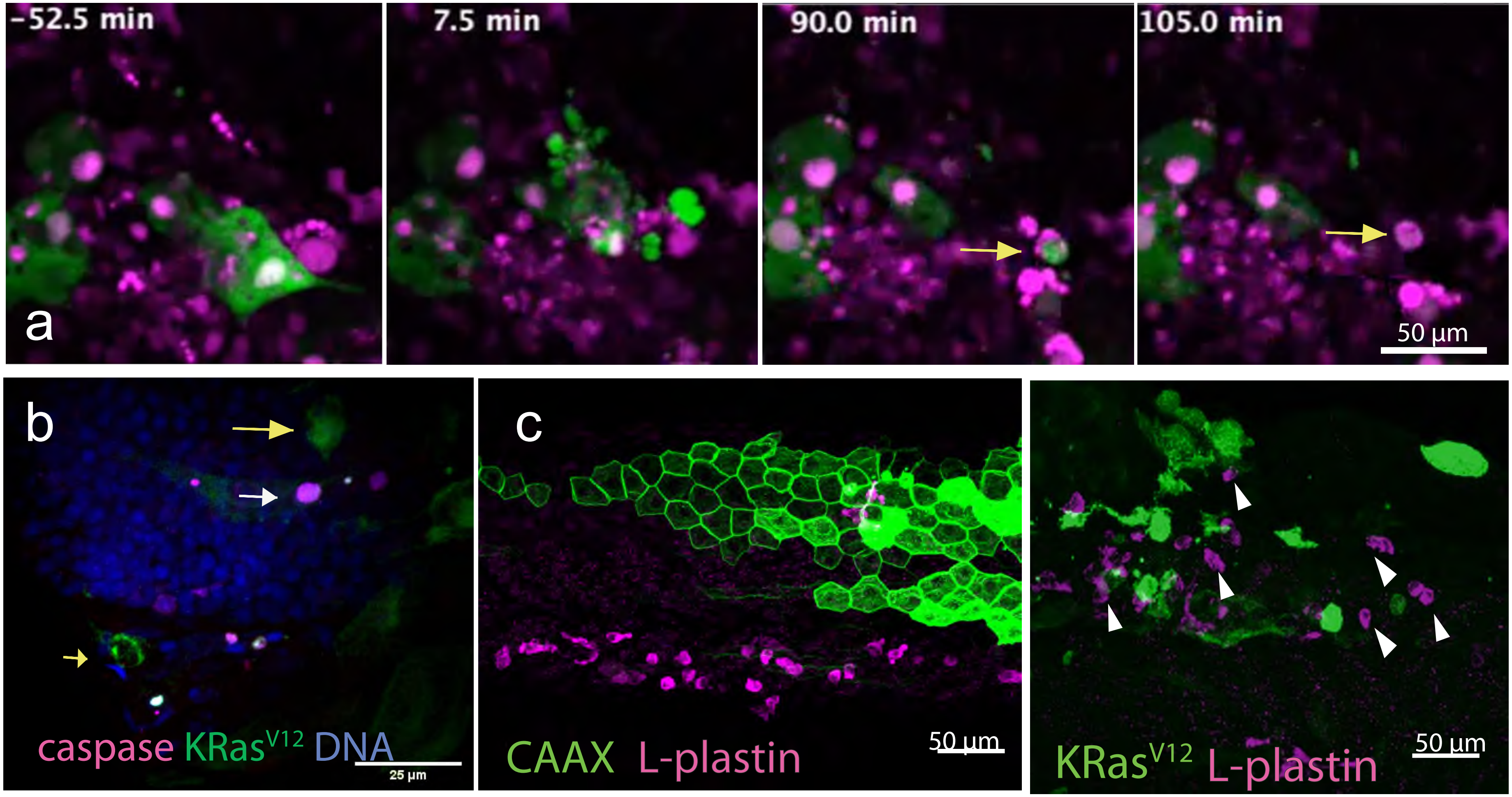

Supplementary Figure 2. Invading cells that undergo apoptosis are rapidly engulfed whereas those surviving are not engulfed by macrophages. (a) Stills from Supplementary Movie 7 showing a dying T2A-KRas^{V12} cell (green), fragmenting, where one fragment is engulfed by another cell (magenta, yellow arrows) within 15'. The embryo is approximately 40hpf at the beginning of the movie, the time after cell death and fragmentation is indicated. (b) EGFP-T2A-KRas^{V12} cells positive (white arrowhead) or negative (yellow arrowhead) for caspase-3, 48 hpf. (c) Macrophages (L-plastin) do not colocalize with CAAX or dt-KRas cells, where only cells that have not invaded are highlighted with a dotted line, 48 hpf. Source data are provided as a Source Data file.

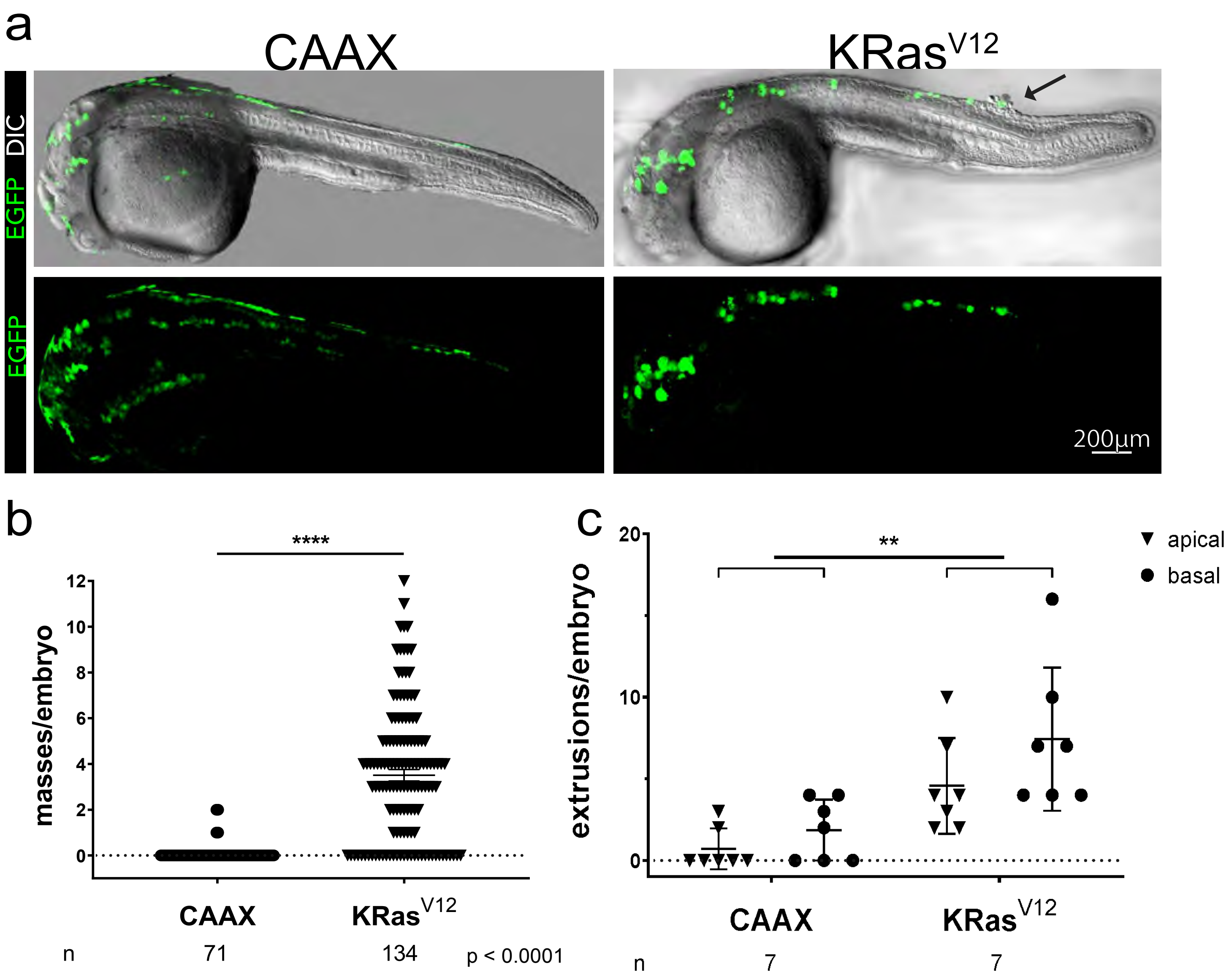

Supplementary Fig. 3. Loss of p53 enhances cell mass formation and extrusion rates of KRas^{V12} cells. (a) 24 hpf p53mut embryos expressing EGFP-CAAX or dt-KRas^{V12}, with arrow pointing to mass. (b) Quantification of cell masses per embryo for CAAX- versus dt-KRas^{V12}-injected in p53^{mut} and p53^{MO} embryos ± SEM, *** $P < 0.001$ by a Mann–Whitney test, n=embryos. (c) Number of apical and basal extrusions per embryo in EGFP-CAAX versus dt-KRas^{V12} p53^{MO} embryos expressed mean ± SEM, ** $P < 0.01$ by a Mann-Whitney test for apical and basal extrusions pooled together, n=embryos. Source data are provided as a Source Data file.

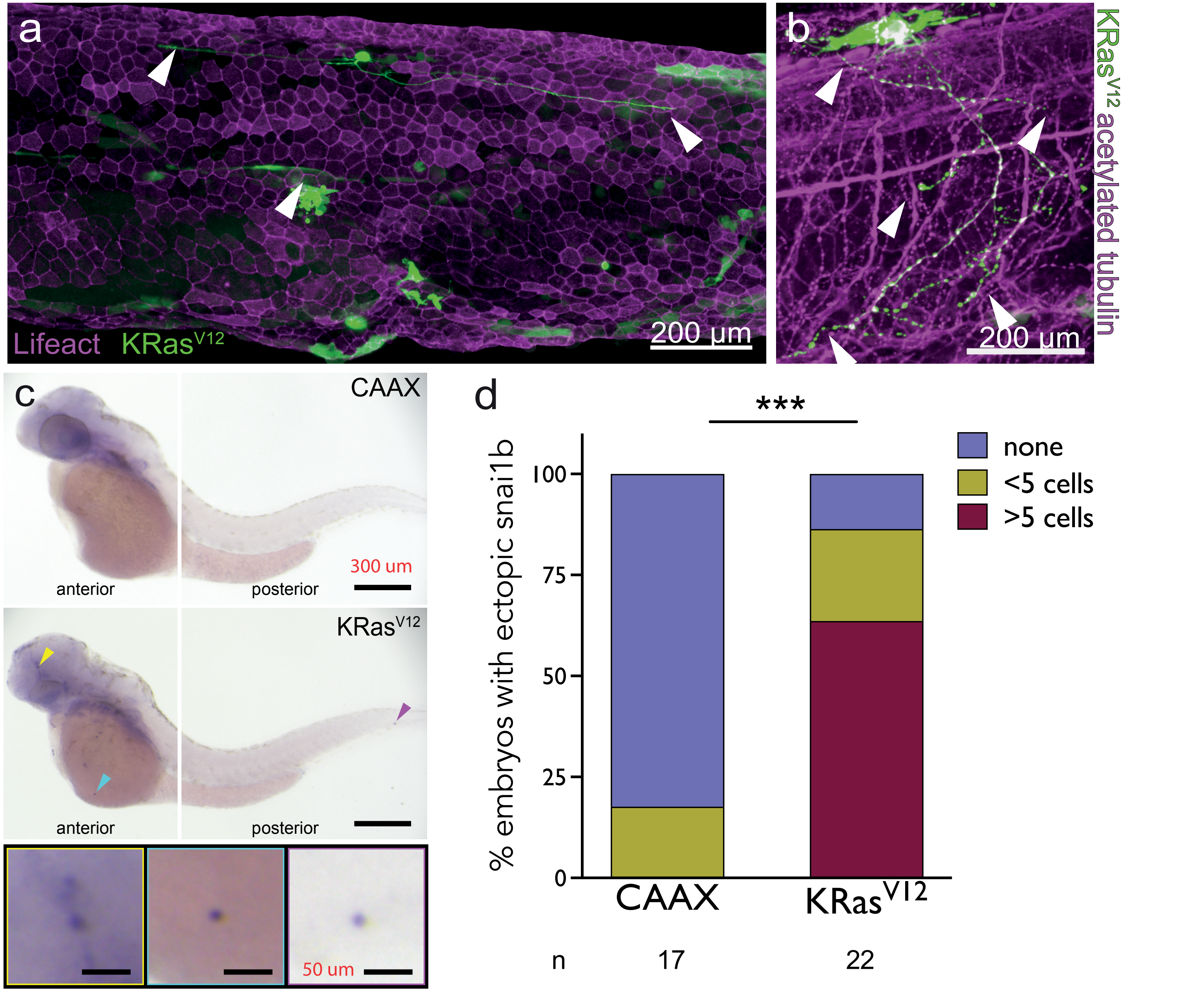

Supplementary Fig. 4. Characterization of invaded cells. (a) A KRT4-T2A-KRas^{V12} cell with neuron-like morphology in a periderm:Lifeact-mCherry reporter line, with arrowheads indicating long processes extending bi-directionally. (b) Acetylated tubulin (AcTub) immunostaining of neuron-like KRT4-T2A-KRas^{V12} cell with arrowheads indicating colocalization, 48 hpf. (c) Whole-mount in situ hybridization detection of the snai1b transcript with arrowheads indicating endogenously-expressed snai1b+ cells, with colored zoomed insets. (d) Percentage of T2A-KRas^{V12}- versus CAAX-expressing embryos with snai1b+ ectopic cells, *** $P < 0.001$ by a Fisher Exact test. All zebrafish are 48 hpf.Source data are provided as a Source Data file

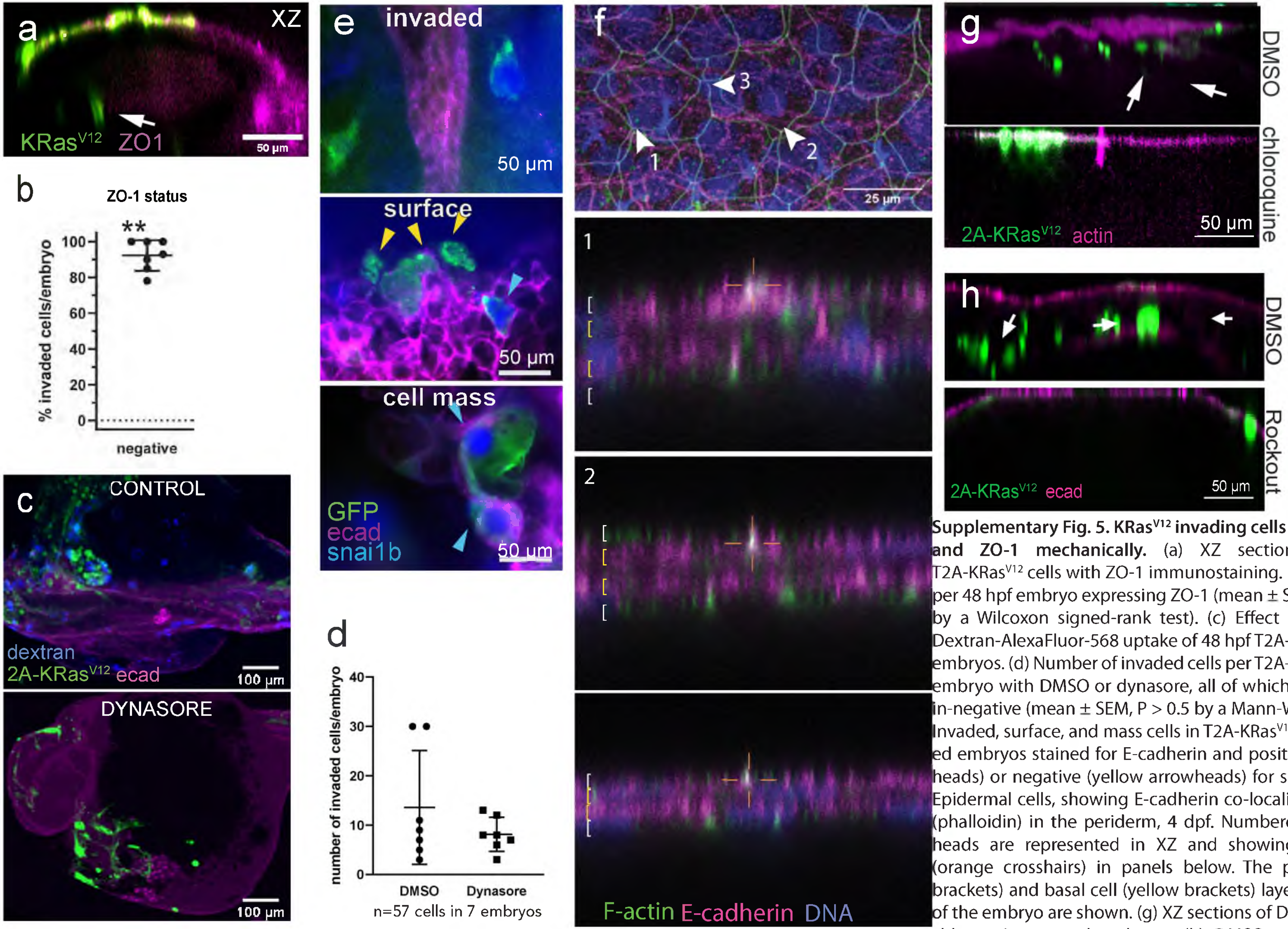

Supplementary Fig. 5. KRas^{V12} invading cells lose E-cadherin and ZO-1 mechanically. (a) XZ sections of invaded T2A-KRas^{V12} cells with ZO-1 immunostaining. (b) Invaded cells per 48 hpf embryo expressing ZO-1 (mean ± SEM, ** $P = 0.016$ by a Wilcoxon signed-rank test). (c) Effect of dynasore on Dextran-AlexaFluor-568 uptake of 48 hpf T2A-KRas^{V12}-injected embryos. (d) Number of invaded cells per T2A-KRas^{V12}-injected embryo with DMSO or dynasore, all of which were E-cadherin-negative (mean ± SEM, $P > 0.5$ by a Mann-Whitney test). (e) Invaded, surface, and mass cells in T2A-KRas^{V12}/p53MO-injected embryos stained for E-cadherin and positive (cyan arrowheads) or negative (yellow arrowheads) for snai1b mRNA. (f) Epidermal cells, showing E-cadherin co-localizes with F-actin (phalloidin) in the periderm, 4 dpf. Numbered white arrowheads are represented in XZ and showing colocalization (orange crosshairs) in panels below. The periderm (white brackets) and basal cell (yellow brackets) layers of both sides of the embryo are shown. (g) XZ sections of DMSO- or 500 µM chloroquine-treated embryos. (h) DMSO- or 50 µM Rockout-treated embryos (with arrows pointing to invaded cells), quantified in Fig. 4, All zebrafish are 48 hpf. Source data are provided as a Source Data file.

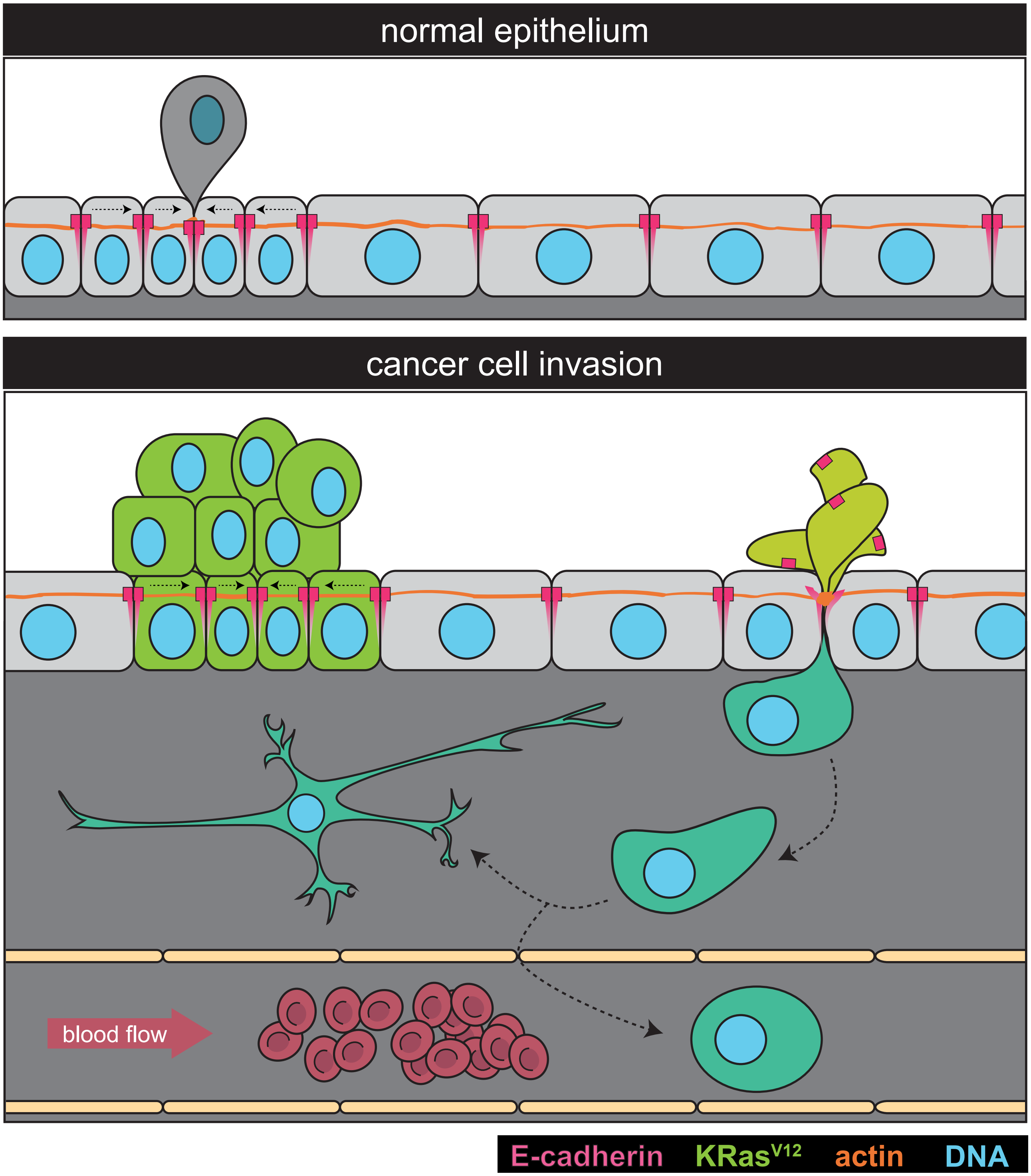

Supplementary Fig. 6. Model. Top panel: In wild-type epithelium, cells get extruded and die when they become too crowded. **Bottom panel:** Epithelia with an oncogenic mutation in KRas form masses at crowded sites (left) or invade under the epithelium by basal cell extrusion (BCE) at completely separate sites (right). BCE allows cells to invade and simultaneously pinch off their apical epithelial determinants, enabling migration, new plasticity, proliferation, and entry into bloodstream. While KRas-transformation enables invasion, most cells will die unless p53 is also mutated.

Supplementary Table 1: Antibodies used within the study

Antibodies used in this study	MANUFACTURER	IDENTIFIER	VALIDATION
Chicken α-GFP	Abcam	Cat. # ab13970<br>RRID: AB_300798	The manufacturer's website (https://www.abcam.com/GFP-antibody-ab13970.html) currently lists 2506 publications that have used this antibody.
Rabbit α-Tp63	GeneTex	Cat. # GTX124660<br>RRID: AB_11175363	The manufacturer's website (https://www.genetex.com/Product/Detail/Tp63-antibody/GTX124660#references) currently lists 8 publications that have used this antibody, 6 of which demonstrate immunohistochemical reactivity in zebrafish.
Mouse α-E-cadherin	BD Biosciences	Cat. # 610181<br>RRID: AB_397580	The manufacturer's website (https://www.bdbiosciences.com/en-us/products/reagents/microscopy-imaging-reagents/immunofluorescence-reagents/purified-mouse-anti-e-cadherin.610181) currently lists 5 publications that have used this antibody.<br><br>The ZFIN antibody database (http://zfin.org/ZDB-ATB-100728-10) currently lists 49 publications that have used this antibody.
Mouse α-N-cadherin	BD Biosciences	Cat. # 610920<br>RRID: AB_2077527	The manufacturer's website (https://www.bdbiosciences.com/en-us/products/reagents/microscopy-imaging-reagents/immunofluorescence-reagents/purified-mouse-anti-n-cadherin.610920) currently lists 5 publications that have used this antibody.
Mouse α-ZO-1	Thermo Fisher Scientific	Cat. # 33-9100<br>RRID: AB_2533147	The manufacturer's website (https://www.thermofisher.com/antibody/product/ZO-1-Antibody-clone-ZO1-1A12-Monoclonal/33-9100) currently lists 547 publications that have used this antibody.<br><br>The ZFIN antibody database (http://zfin.org/ZDB-ATB-081105-5) currently lists 190 publications that have used this antibody.
Mouse α-acetylated tubulin	Sigma-Aldrich	Cat. # T6793<br>RRID: AB_477585	The manufacturer's website (https://www.sigmaaldrich.com/GB/en/product/sigma/t6793) currently lists 1076 publications that have used this antibody.<br><br>The ZFIN antibody database (http://zfin.org/ZDB-ATB-081003-6) currently lists 540 publications that have used this antibody.
Rabbit α-caspase-3	BD Biosciences	Cat. # 559565<br>RRID: AB_397274	The manufacturer's website (https://www.bdbiosciences.com/en-us/products/reagents/flow-cytometry-reagents/research-reagents/single-color-antibodies-ruo/purified-rabbit-anti-active-caspase-3.559565) currently lists 3 publications that have used this antibody.<br><br>The ZFIN antibody database (http://zfin.org/ZDB-ATB-081107-1) currently lists 101 publications that have used this antibody.
Sheep α-digoxigenin-alkaline phosphatase, Fab fragments	Roche	Cat. # 11093274910<br>RRID: AB_514497	The manufacturer's website (https://www.sigmaaldrich.com/GB/en/product/roche/11093274910) currently lists 373 publications that have used this antibody.

Goat α-chicken IgY AlexaFluor-488	Thermo Fisher Scientific	Cat. # A11039<br>RRID: AB_2534096	Secondary antibody
Goat α-rabbit IgG AlexaFluor-568	Thermo Fisher Scientific	Cat. # A11011<br>RRID: AB_143157	Secondary antibody
Goat α-mouse IgG AlexaFluor-647	Thermo Fisher Scientific	Cat. # A21235<br>RRID: AB_2535804	Secondary antibody
