# Supplementary material for: KRas-transformed epithelia cells invade and partially dedifferentiate by basal cell extrusion
Source: Nat Commun. 2021 Dec 10;12:7180. doi: 10.1038/s41467-021-27513-z (PMC8664939; doi:10.1038/s41467-021-27513-z)
Supplement: Supplementary file 3 — Description of Additional Supplementary Files [file 41467_2021_27513_MOESM3_ESM.pdf]

Description of Additional Supplementary Files

Supplementary Movie 1. *krt4*:EGFP-CAAX cells remain in the epidermis without extruding (hh:mm).

Supplementary Movie 2. *krt4*:EGFP-dt-KRas^{V12} cell apically extruding (hh:mm).

Supplementary Movie 3. *krt4*:EGFP-dt-KRas^{V12} cell basally extruding (hh:mm).

Supplementary Movie 4. Unusual (only one found) EGFP-T2A-KRas^{V12} cells (green) migrating away from an internalized mass in a periderm:Lifeact-mCherry reporter (magenta) (hh:mm), with arrow marking migrating cell.

Supplementary Movie 5. Rotating volume view of an EGFP-dt-KRas^{V12} cell (green) internalized between two p63^{+} (magenta) basal cell layers.

Supplementary Movie 6. *krt4*:mCherry-T2A-cMyc-expressing peridermal cells (magenta) in a in a *cldn*:lyn-EGFP reporter line (green) do not form masses or extrude.

Supplementary Movie 7. An EGFP-dt-KRas^{V12} cell (green) expressing *H2B-RFP* (magenta) dies shortly after invasion, fragments and becomes engulfed by neighboring GFP-negative cell. (hh:mm).

Supplementary Movie 8. A basally extruding, invading, and migrating EGFP-T2A-KRas^{V12} epidermal cell (green) in a periderm:Lifeact-mCherry reporter (magenta). The migration path is traced in a dashed yellow line (hh:mm).

Supplementary Movie 9. An EGFP-dt-KRas^{V12} cell (green) in a *h2afva*:h2afva-mCherry reporter (magenta) invades by BCE and migrates away (hh:mm).

Supplementary Movie 10. An EGFP-T2A-KRas^{V12} cell (green) in a mpeg:mCherry reporter of macrophages, showing no interaction between the two cell types.

Supplementary Movie 11. An EGFP-dt-KRas^{V12} cell (green) in a *h2afva*:h2afva-mCherry reporter (magenta) pauses migration, divides, and the daughter cells migrate away from each other (hh:mm).

Supplementary Movie 12. Zoom of a movie showing an EGFP-dt-KRas^{V12} cell (green) stuck within the blood vessel of a *kdrl:*mCherry reporter (magenta) (hh:mm).

Supplementary Movie 13. Zoom of a movie showing an EGFP-dt-KRas^{V12} cell (green) stuck within the blood vessel of a *kdrl:*mCherry reporter (magenta) (hh:mm).

Supplementary Movie 14. Zoom of a movie showing an EGFP-dt-KRas^{V12} cell (green) impedes blood flow in a *gata1a:*mCherry reporter (magenta) (hh:mm).

Supplementary Movie 15. An EGFP-T2A-KRas^{V12} cell (green) in a periderm:Lifeact-mCherry reporter (magenta) with bipolar projections, similar to a neuron (hh:mm).

Supplementary Movie 16. High-resolution time-lapse imaging of a basally extruding EGFP-dt-KRas^{V12} cell (green) in a periderm:Lifeact-mCherry reporter (magenta). Numerous blebs form as the actomyosin cable constricts and the apical membrane pinches off (hh:mm).

Supplementary Movie 17. Low-resolution time-lapse imaging of a basally extruding EGFP-dt-KRas^{V12} cell (green) in a periderm:NTR-mCherry reporter (red), where cell blebs during BCE and loses green before migrating away as a red cell (hh:mm).

Supplementary Movie 18. Low-resolution time-lapse imaging of a basally extruding EGFP-dt-KRas^{V12} cell (green) in a periderm:NTR-mCherry reporter (red), where cell blebs during BCE and loses green before migrating away as a red cell (hh:mm).

Supplementary Movie 19.

High-resolution movie focusing through Z-slices projected in Supplementary Fig. 5F. F-actin (green), E-cadherin (magenta), DNA (blue).
