# Supplementary material for: KRas-transformed epithelia cells invade and partially dedifferentiate by basal cell extrusion
Source: Nat Commun. 2021 Dec 10;12:7180. doi: 10.1038/s41467-021-27513-z (PMC8664939; doi:10.1038/s41467-021-27513-z)
Supplement: Supplementary file 23 — Reporting Summary [file 41467_2021_27513_MOESM23_ESM.pdf]

nature research

Corresponding author(s): Rosenblatt, Jody

Last updated by author(s): 15/11/2021

Reporting Summary

Nature Research wishes to improve the reproducibility of the work that we publish. This form provides structure for consistency and transparency in reporting. For further information on Nature Research policies, see our Editorial Policies and the Editorial Policy Checklist.

Statistics

For all statistical analyses, confirm that the following items are present in the figure legend, table legend, main text, or Methods section.

n/a	Confirmed	
☐	☒	The exact sample size (*n*) for each experimental group/condition, given as a discrete number and unit of measurement
☐	☒	A statement on whether measurements were taken from distinct samples or whether the same sample was measured repeatedly
☐	☒	The statistical test(s) used AND whether they are one- or two-sided<br>*Only common tests should be described solely by name; describe more complex techniques in the Methods section.*
☒	☐	A description of all covariates tested
☐	☒	A description of any assumptions or corrections, such as tests of normality and adjustment for multiple comparisons
☐	☒	A full description of the statistical parameters including central tendency (e.g. means) or other basic estimates (e.g. regression coefficient) AND variation (e.g. standard deviation) or associated estimates of uncertainty (e.g. confidence intervals)
☐	☒	For null hypothesis testing, the test statistic (e.g. *F*, *t*, *r*) with confidence intervals, effect sizes, degrees of freedom and *P* value noted<br>*Give P values as exact values whenever suitable.*
☒	☐	For Bayesian analysis, information on the choice of priors and Markov chain Monte Carlo settings
☒	☐	For hierarchical and complex designs, identification of the appropriate level for tests and full reporting of outcomes
☒	☐	Estimates of effect sizes (e.g. Cohen's *d*, Pearson's *r*), indicating how they were calculated

Our web collection on statistics for biologists contains articles on many of the points above.

Software and code

Policy information about availability of computer code

Data collection	NIS Elements Advanced Research v4.60 and v.5.30, Leica Application Suite X v3.4.2.18368, National Instruments LabVIEW 2012
Data analysis	Fiji (ImageJ 1.52p), GraphPad Prism v8, Matlab 2019b, mSPIM data processing tools (Daetwyler et al., 2019)

For manuscripts utilizing custom algorithms or software that are central to the research but not yet described in published literature, software must be made available to editors and reviewers. We strongly encourage code deposition in a community repository (e.g. GitHub). See the Nature Research guidelines for submitting code & software for further information.

Data

Policy information about availability of data

All manuscripts must include a data availability statement. This statement should provide the following information, where applicable:

- Accession codes, unique identifiers, or web links for publicly available datasets
- A list of figures that have associated raw data
- A description of any restrictions on data availability

Associated raw data are available from the corresponding author upon request, due to the fact that movies were made from a variety of different labs, saved on different servers, and require over 20 Tb of space.

Field-specific reporting

Please select the one below that is the best fit for your research. If you are not sure, read the appropriate sections before making your selection.

☒ Life sciences ☐ Behavioural & social sciences ☐ Ecological, evolutionary & environmental sciences

For a reference copy of the document with all sections, see nature.com/documents/nr-reporting-summary-flat.pdf

Life sciences study design

All studies must disclose on these points even when the disclosure is negative.

Sample size	The minimum sample size was determined according to the standards in the field and based on these calculations: Charan, Jaykaran, and N D Kantharia. "How to calculate sample size in animal studies?." Journal of pharmacology & pharmacotherapeutics vol. 4,4 (2013): 303-6. doi:10.4103/0976-500X.119726
Data exclusions	Notochord and muscle cells, as well as melanocytes, that misexpressed the GFP-tagged proteins were excluded from analyses as these are known artifacts of F0 transgenics in zebrafish (Mosimann et al., Dev Dyn 2013).
Replication	Experiments were performed at least twice with similar results for all trials.
Randomization	One-cell embryos were randomly chosen from the clutch to be injected. Injected embryos were similarly assigned randomly to experimental groups after sorting for expression of fluorescent transgenes.
Blinding	Blinding was not possible for this study as CAAX- and KRasV12-injected embryos are very distinguishable from each other.

Reporting for specific materials, systems and methods

We require information from authors about some types of materials, experimental systems and methods used in many studies. Here, indicate whether each material, system or method listed is relevant to your study. If you are not sure if a list item applies to your research, read the appropriate section before selecting a response.

Materials & experimental systems

n/a	Involved in the study
☐	☒ Antibodies
☒	☐ Eukaryotic cell lines
☒	☐ Palaeontology and archaeology
☐	☒ Animals and other organisms
☒	☐ Human research participants
☒	☐ Clinical data
☒	☐ Dual use research of concern

Methods

n/a	Involved in the study
☒	☐ ChIP-seq
☒	☐ Flow cytometry
☒	☐ MRI-based neuroimaging

Antibodies

Antibodies used	Please see attached sheet on antibodies used in this study.
Validation	Please see attached sheet on antibodies used in this study.

Animals and other organisms

Policy information about studies involving animals; ARRIVE guidelines recommended for reporting animal research

Laboratory animals	AB wild-type and tp53(zdf1/zdf1) (Berghmans et al., PNAS 2005) mutant zebrafish lines were used in this study. Enhancer trap lines were generated in the lab of Richard Dorsky (Otsuna et al., Dev Dyn 2015) and characterized in our lab (Eisenhoffer et al., J Cell Sci 2017). Adult zebrafish were used only for breeding. Embryos and larvae were used until 5 dpf at ages indicated in the figures or text. At these developmental stages, sexual characteristics have not yet manifested.
Wild animals	No wild animals were used in this study.
Field-collected samples	No field collected animals were used for this study.
Ethics oversight	University of Utah - Centralized Zebrafish Animal Resource: All zebrafish procedures adhered to the Institutional Animal Care and Use Committee guidelines in compliance with the US Animal Welfare Act. King's College London - Biological Services Unit: All zebrafish procedures adhered to the Animals in Scientific Procedures Act 1986 and animal use guidelines of the UK Home Office.

Note that full information on the approval of the study protocol must also be provided in the manuscript.
